# Combination Antiretroviral Therapy for HIV in Rwandan Adults: Clinical Outcomes and Impact on Reproductive Health up to 24 Months

**DOI:** 10.1155/2015/740212

**Published:** 2015-07-16

**Authors:** Brenda Asiimwe-Kateera, Nienke Veldhuijzen, Jean Paul Balinda, John Rusine, Sally Eagle, Joseph Vyankandondera, Julie Mugabekazi, Pascale Ondoa, Kimberly Boer, Anita Asiimwe, Joep Lange, Peter Reiss, Janneke van de Wijgert

**Affiliations:** ^1^INTERACT Program, Kigali, Rwanda; ^2^College of Medicine and Health Sciences, University of Rwanda, Kigali, Rwanda; ^3^Academic Medical Center (AMC), Department of Global Health and Amsterdam Institute for Global Health and Development (AIGHD), 1105 BM Amsterdam, Netherlands; ^4^Department of Epidemiology and Biostatistics, VU University Medical Center, 1007 MB Amsterdam, Netherlands; ^5^Treatment and Research for AIDS Center (TRAC-Plus), Kigali, Rwanda; ^6^National Reference Laboratory, Kigali, Rwanda; ^7^Institute of Infection and Global Health, University of Liverpool, Liverpool L69 7BE, UK; ^8^Kigali University Teaching Hospital, Kigali, Rwanda; ^9^Rinda Ubuzima, Kigali, Rwanda; ^10^Biomedical Research, Royal Tropical Institute, 1105 AZ Amsterdam, Netherlands; ^11^Ministry of Health of Rwanda, Rwanda

## Abstract

Adult women (*n* = 113) and men (*n* = 100) initiating combination antiretroviral therapy (cART) and women not yet eligible for cART (*n* = 199) in Kigali, Rwanda, were followed for 6–24 months between 2007 and 2010. In the cART groups, 21% of patients required a drug change due to side effects and 11% of patients had virological failure (defined as >1,000 HIV RNA copies/mL) after 12 months of cART. About a third of the pregnancies since HIV diagnosis were unintended. The proportion of women in the pre-cART group using modern contraception other than condoms (50%) was similar to women in the general population, but this proportion was only 25% in women initiating cART. Of the women who carried at least one pregnancy to term since having been diagnosed HIV-positive, a third reported to have participated in a prevention-of-mother-to-child-transmission (PMTCT, option A) intervention. Many patients were coinfected with herpes simplex virus type 2 (79–92%), human papillomavirus (38–53%), and bacterial sexually transmitted infections (STIs) with no differences between groups. We applaud the Rwandan government for having strengthened family planning and PMTCT services and for having introduced HPV vaccination in recent years, but additional work is needed to strengthen STI and HPV-related cancer screening and management in the HIV-positive population.

## 1. Introduction

Rwanda has had a sustained general adult HIV prevalence of 3.0% countrywide and 7.0% in the capital city Kigali over the last 10 years [1]. However, the HIV prevalence is much higher in vulnerable populations. The 2007/2008 Kigali HIV Incidence Study (KHIS) found an HIV prevalence of 24% in female sex workers and 13% in female clients of HIV counseling and testing (HCT) sites [2, 3]. Thanks to the Rwandan government and international efforts, access to combination antiretroviral therapy (cART) has drastically improved from only 870 adults receiving cART in 2002 to 114,995 in 2013, which is more than 90% of the eligible HIV-infected population using the 350 cells/*μ*L threshold [4]. Rwanda has passed the “tipping point” beyond which the number of people starting HIV treatment exceeds the number of people acquiring HIV infection. Even though access to plasma HIV viral load testing is limited or absent and cART efficacy is typically monitored clinically and by regular assessment of CD4+ T-cell counts, the short term outcomes of cART in Rwanda and other resource-constrained settings have been shown to be comparable to those in resource-rich settings [5–8]. However, this requires regular monitoring due to the emergence of HIV drug resistance and other epidemic and programmatic changes.

Rwanda has also made significant gains in family planning in the last decade. The government launched an ambitious family planning program in 2006. As a result, the percentage of women using a modern contraceptive method (including condoms) increased from only 10% of currently married women in 2005 to 45% in 2010, and the total fertility rate dropped from 6.1 children per woman in 2005 to 4.2 in 2010 [9]. However, unmet need for family planning has not yet been eliminated [9].

In contrast, Rwanda has placed much less emphasis on the control of other sexually transmitted infections (STIs) in the last decade. Diagnostic testing for STIs, with the exception of syphilis, is not readily available, and clinicians are taught to use syndromic management, which is known to be suboptimal [10, 11]. Data on the prevalence of STIs in Rwanda are scarce. In the above-mentioned KHIS study, the herpes simplex virus type 2 (HSV-2) prevalence was 60% in female sex workers and 43% in female HCT clients. Only the sex workers were tested for additional STIs and all of these were also common (high risk human papillomavirus (HR-HPV) 36%, syphilis 7%, gonorrhea 12%, chlamydia 5%, and trichomoniasis 17%) [2, 3, 12]. Coinfection with STIs is known to increase genital HIV viral loads, potentially resulting in increased onward HIV transmission [13]. Furthermore, coinfection of HIV and HR-HPV types is known to increase the likelihood of development of cervical and other HPV-related cancers [14].

The effects of chronic HIV infection and cART on sexual and reproductive health have not yet been widely investigated. A recent study in Uganda found that pregnancy incidence in HIV-positive women in routine care was comparable to the incidence in the general population and that cART use was not a significant predictor of pregnancy incidence in HIV-positive women [15]. Analysis of Demographic and Health Surveys (DHS) data from 9 sub-Saharan countries showed that HIV status did not significantly change fertility intentions or contraceptive use in most countries, except Rwanda, where HIV-positive women who were aware of their status had lower odds of wanting more children and were more likely to use contraception [16]. A study in Kenya also found that HIV-positive women had lower fertility intentions, but in that study, their contraceptive use was similar to that of HIV-negative women [17].

The SEARCH study (Side Effects and Reproductive Health in a Cohort on HAART) was a prospective cohort study in Kigali, Rwanda, aimed at evaluating cART adherence and clinical outcomes and the impact of cART on various aspects of sexual and reproductive health. The cART adherence data were reported elsewhere [18]. Briefly, adherence by pill count was only about 50% in the first 3 months of cART, increasing to between 70% and 80% from months 3 to 12. In this paper, we focus on the clinical and sexual and reproductive health outcomes.

## 2. Materials and Methods

The SEARCH study was conducted from November 2007 until August 2010 at the HIV outpatient clinic of the Center for Treatment and Research on AIDS, Tuberculosis and Malaria (TRAC-Plus), which is now part of the Institute of HIV/AIDS, Disease Prevention and Control (IHDPC) within the Rwanda Biomedical Center. Ethical approval was obtained from the Rwanda National Ethics Committee. All study participants provided written informed consent prior to enrollment, were free to withdraw from the study at any time, and continued to receive care within publicly funded HIV treatment programs at the end of their study participation. They were given an equivalent of 3 USD in local currency for each scheduled study visit to compensate them for transport cost and time spent at the clinic.

### 2.1. Study Design and Population

The SEARCH study was designed to enroll 100 women and 100 men initiating cART (referred to as the cART groups) and 200 women who did not yet qualify for cART (referred to as the pre-cART group), according to the Rwanda Ministry of Health Guidelines during the study period [19]. The main purpose of the latter group was to compare reproductive health outcomes between women pre-cART and on cART. The clinical cART eligibility criteria at that time included having a CD4 count below 350 cells/*μ*L and/or WHO clinical stage IV regardless of CD4 count [19]. Furthermore, the national guidelines called for several social eligibility criteria, such as having disclosed one's HIV status to at least one family member and being willing to involve a relative or friend in the cART process to facilitate adherence [19]. The decision to start a patient on cART was made by a clinic committee consisting of clinicians and social workers. For the cART groups, only patients deemed eligible for cART by this committee were approached by a physician for potential participation in the SEARCH study. For the pre-cART group, physicians approached women who were not yet eligible for cART initiation because their CD4 count was higher than 350 cells/*μ*L. In all groups, patients were excluded from study participation if they were younger than 18 years, had used cART in the past (with the exception of participation in a prevention-of-mother-to-child-transmission (PMTCT) program; only PMTCT option A was available in Rwanda during the study), were clinically unstable, had confirmed or suspected tuberculosis, or were already participating in other research studies.

During the study period, decentralization of HIV care in Rwanda had already begun but was still in the early stages; the TRAC-Plus Clinic was one of the largest HIV clinics in Kigali and was treating HIV patients from all over the city. All eligible patients attending the TRAC-Plus Clinic between November 2007 and January 2010 were offered enrollment into the study. To improve enrollment rates, some patients from a nearby public clinic (Gitega clinic) were actively referred to the TRAC-Plus Clinic until enrollment was completed. Patients were followed up for 6 to 24 months, until the study was closed in August 2010. The study visit schedule followed the national ART program schedule as much as possible so that study participants could easily be transferred from SEARCH to the national ART program when the study ended. Women not eligible for cART were seen at baseline and 3-month intervals thereafter, and they were switched to the cART group when they became eligible for ART. For patients initiating cART, clinic visits were scheduled at baseline (cART initiation), week 2, and months 1, 2, 3, 6, 9, and 12 after cART initiation. In addition, pharmacy visits were scheduled monthly.

### 2.2. Study Procedures

All participants were interviewed regularly throughout the study about sociodemographics, sexual and contraceptive behavior, HIV and reproductive history, cART adherence, and symptoms (with a focus on those potentially related to HIV infection, cART use, or reproductive health problems). Side effects, perceived overall weight changes, and perceived fat redistribution were also assessed by structured questionnaires at regular intervals but only in women and men on cART. Clinical assessments were carried out every 6 months and when clinically indicated and included a speculum and bimanual exam in women and a targeted assessment for sensory neuropathy in women and men.

Blood samples were collected at all clinic visits. They were hand-carried from the clinic to the laboratory next door within 4 hours after collection. CD4+ T-cell counts on EDTA blood samples were always done on the day of sample collection. All other blood samples were stored as aliquots of plasma or serum at −80°C until further processing and testing. Endocervical specimens were collected every 6 months during pelvic exams. A swab was vigorously agitated in Amplicor specimen transport medium (Roche Molecular Systems, Branchburg, NJ, USA), and a cytobrush in PreservCyt medium (Hologic, Bedford, MA, USA). In men, the penile glans, sulcus corona, shaft, and scrotum were swabbed using saline wetted swabs, which were also agitated in PreservCyt medium. All genital specimens were stored at −80°C.

All laboratory tests were carried out at the National Reference Laboratory in Kigali, Rwanda, except for HPV testing (see below). At study enrollment, the presence of HIV infection in each study participant was confirmed using a 4th generation HIV ELISA. CD4+ T-cell counts (FACSCalibur, Becton Dickinson, San Jose, CA, USA) were done every 3 months for women who did not yet qualify for cART and every 6 months for those initiating cART. Plasma HIV RNA viral loads (COBAS AmpliPrep/COBAS TaqMan HIV-1 Test versions 2.0, Roche Molecular Diagnostics, Pleasanton, CA, USA) were done at cART initiation and every 12 months thereafter. The lower limit of detection was 40 HIV RNA copies/mL. Women were tested for pregnancy using an hCG urine dipstick test at baseline and every 6 months. Participants were tested for herpes simplex type 2 (HSV-2) using HerpeSelect test kits (Focus Diagnostics, Cypress, CA, USA) at baseline, for syphilis by RPR confirmed by TPHA (Human Diagnostics, Wiesbaden, Germany) at baseline and every 6 months, and for* Neisseria gonorrhoeae* and* Chlamydia trachomatis* by PCR (COBAS Amplicor, Roche Molecular Systems, Branchburg, NJ, USA) at baseline and (women only) every 12 months. HPV genotyping on PreservCyt specimens was performed at the Department of Pathology at the VU University Medical Center in Amsterdam, Netherlands. Duplicate GP5+/6+ PCR enzyme immunoassays followed by reverse line blot analysis on positive samples were carried out as described previously [20]. A mixture of PCR probes was used for the detection of 14 HR-HPV types (HPV16, 18, 31, 33, 35, 39, 45, 51, 52, 56, 58, 59, 66, and 68). Data on hepatitis B, hepatitis C, alanine aminotransferase (ALT), and aspartate aminotransferase (AST) in this cohort were reported elsewhere [21].

Participants were treated for curable STIs by syndromic management in accordance with the national guidelines in Rwanda [10]. If a laboratory test result came back positive and syndromic treatment had not yet been given, women were recalled to the clinic for treatment. Partner notification and treatment were offered for laboratory-confirmed infections.

### 2.3. Statistical Analysis

Data were analyzed using R version 2.12.1 (The R Project for Statistical Computing, http://www.r-project.org/). Binary and categorical data were summarized as proportions and continuous data as medians and interquartile ranges (IQR) or ranges. We only did pairwise comparisons, either comparing women pre-cART to women and men on cART (cART outcomes), women pre-cART to women on-cART (reproductive health outcomes), women on cART to men on cART (cART side effects), or month 6 to month 12 data within one study group, as indicated in the text and footnotes of the tables. Logistic regression was used for binary variables, Chi-square tests for categorical variables, and quasi-Poisson general linear models (GLM) for medians. Comparisons that are not explicitly indicated as statistically significant in the text or tables were not significant at the *p* < 0.05 level. STI incidence rates were calculated per 100 person years of follow-up (PY) with 95% confidence intervals (95% CI). Pregnancy and HSV-2 were censored at the first positive laboratory test result.

## 3. Results

### 3.1. Participant Disposition

**Figure 1:**
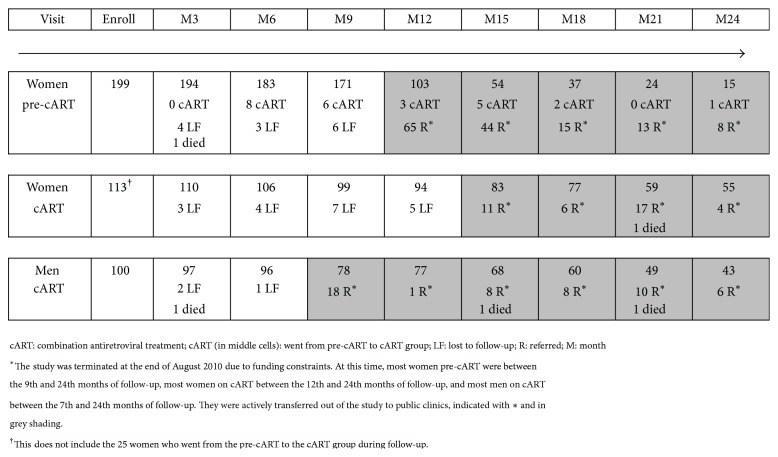
Participants flow.

One hundred thirteen women and 100 men were enrolled in the cART groups and 199 women in the pre-cART group.  Figure 1 shows the participant flow in each of the 3 groups. Funding ended at the end of August 2010, prompting active referral from the study clinic to public clinics to ensure continuation of cART treatment. Retention rates were 86%, 83%, and 96% prior to August 2010 in women pre-cART, women initiating cART, and men initiating cART, respectively, but dropped thereafter mainly due to these active referrals (the active referral period is indicated in grey shading in Figure 1). Five participants died (one in each of the women's groups and 3 in the male group).

Baseline sociodemographic characteristics are shown in Table 1. The men initiating cART had known for a median of one year that they were HIV-positive, whereas the women in both the cART and pre-cART groups had known for a median of two years (Table 1). Of those who were married, 77% in each group had a spouse who was also HIV-positive.

### 3.2. cART Regimens and Clinical Efficacy

A combination of zidovudine (AZT), lamivudine (3TC), and nevirapine (NVP) was the first-line regimen of choice in the Rwandan Antiretroviral Treatment Guidelines of 2007, but this was revised to tenofovir (TDF), 3TC, and NVP in the 2009 updated guidelines [19, 22]. Of the 213 women and men initiating cART at baseline, 71% received AZT/3TC/NVP, 19% TDF/3TC/NVP, 5% stavudine (D4T)/3TC/NVP, 3% AZT/3TC/efavirenz (EFV), and 3% other combinations of these drugs. Of the 199 women in the pre-cART group, 25 started cART during study follow-up, and they also mostly received AZT/3TC/NVP or TDF/3TC/NVP.

By design, the women and men in the cART groups had a lower median CD4 count at baseline than the women in the pre-cART group (Table 2). As expected, their median plasma HIV viral load was higher, and they were more likely to be in WHO clinical stages II–IV (Table 2). Also as expected, the median plasma HIV viral load dropped to undetectable levels (<40 HIV RNA copies/mL) in the two cART groups during the first year of cART and remained stable at undetectable levels in the second year, whereas the median plasma viral load of women in the pre-cART group did not significantly change over time (Table 2). The median CD4 count in the two cART groups slowly but steadily increased over time, whereas the median CD4 count in the pre-cART group remained stable in the first year and started declining thereafter. At month 12, virological failure (defined as >1,000 HIV RNA copies/mL) was 11% and immunological failure (defined as failure to achieve a CD4 gain of at least 50 cells above pretherapy levels or having an absolute CD4 count of less than 100 cells/mL after one year of therapy) was 28% [23]. These cases were presented in more detail elsewhere [24].

### 3.3. cART Side Effects and Mental Health

A total of 51 patients had one drug change during study follow-up, and 6 patients had two or more changes. Most of these changes (50; 21% of patients) were in response to drug intolerance or side effects, but 3 regimens were changed when the guidelines were updated in 2009, 2 because of suspected tuberculosis, 2 because of perceived lack of cART efficacy, one because of pregnancy, and 12 for unspecified reasons. While 6% of patients reported to have needed hospitalization for a very severe side effect during the first 6 months of cART, this declined to 2% between months 6 and 12, 1.6% between months 12 and 18, and 1.3% between months 18 and 24. The most commonly self-reported side effects over 24 months of follow-up were mild or moderate nausea, anorexia, muscle weakness, vertigo, fatigue, headache, and insomnia. The reporting of all side effects peaked (to at most 20% of patients) in the first month of cART and declined thereafter without intervention (data not shown). Up to 25% of patients reported an increase in appetite during the first 12 months of cART, but this declined to about 15% at month 24.

Women and men on cART had a significantly higher prevalence of clinically assessed sensory neuropathy at all study visits except month 24 than women pre-cART, but there were no differences between women and men on cART (Table 3). The proportion of patients on cART with sensory neuropathy increased from 18% at baseline to 27% at month 12 and declined thereafter. At month 24, the proportions were similar and low in all three groups. In the 3 months before cART initiation, 38% of women and 49% men reported general weight loss, but this declined to 13–26% at follow-up visits (Table 3). Almost all women and men had noticed no change or fat loss in the face in the 3 months before cART initiation, but 65–80% reported fat gain during follow-up visits (Table 3). Median liver enzyme levels did not change significantly after 12 months of cART, not even when comparing patients with active or past hepatitis B, or hepatitis C, with those who had never had hepatitis B or C [21]. All three groups showed a reduction in feeling depressed between baseline and month 12, but this only reached statistical significance in the two cART groups (Table 4). However, about half the women in both groups and 20% of men still reported regular or frequent feelings of depression at month 12.

### 3.4. Sexual and Reproductive Health at Baseline

About two-thirds of women pre-cART (64%) and men initiating cART (67%) reported at baseline to be currently sexually active compared to about half of the women initiating cART (51%; *p* < 0.05 when compared to women pre-cART) (Table 1). The lifetime number of sex partners was a median of two for women and six for men (*p* < 0.05). About half of the women in each group reported to ever have been forced to have sex against their will. Condom use during the last sex act was 40.6% in women pre-cART, 43.1% in women initiating cART, and 61.2% in men initiating cART (Table 4).

Women in both groups had a median of three lifetime pregnancies (Table 1). More than a third of these pregnancies (38% pre-cART and 46% cART; *p* = 0.22) were unintended and 9% in each group had ended in abortion, miscarriage, or stillbirth. Only a small proportion of women (9.5% pre-cART and 10.8% cART) were actively trying to get pregnant and this did not differ between the groups. While 66% of all women pre-cART and 49% of all women initiating cART said that they used family planning (*p* < 0.05), only 50% and 24% of them, respectively, used a highly effective modern method (hormonal method, IUD, or female sterilization) and most of them also used condoms (data not shown). Twenty-eight percent of women pre-cART and 17% of women initiating cART had had at least one pregnancy since HIV diagnosis: about a third of these in each group were unintended, and 43% and 21% of the women reported to have participated in a PMTCT option A intervention (*p* < 0.05) (Table 1).

Half a percent of women pre-cART and 5% of women initiating cART had a positive pregnancy test at baseline (*p* < 0.05) (Table 2). The majority of women (86% pre-cART and 92% cART) and men (79%) had positive herpes simplex type 2 (HSV-2) serology, and many had HR-HPV by PCR (38%, 53%, and 43%, resp.). Positive diagnostic tests for* Neisseria gonorrhoeae*,* Chlamydia trachomatis*, and syphilis were common, with baseline prevalence rates ranging from 2 to 14% (Table 2). None of these STI prevalence rates were statistically significantly different between groups.

### 3.5. Sexual and Reproductive Health Changes between Baseline and Month 12

Incidence rates of pregnancy,* Neisseria gonorrhoeae*,* Chlamydia trachomatis*, and syphilis were not statistically significantly different between women pre-cART and women on cART, but the numbers of cases were small in both groups (Table 2). Most sexual behavioral characteristics did not change after cART initiation with one exception: men reported a higher frequency of vaginal sex acts after 12 months of cART compared to baseline (Table 4). In the pre-cART group, a smaller proportion of women reported use of family planning at month 12 compared to baseline (49% compared to 66%; *p* = 0.03).

All three groups showed either no decline or improvement in sexual desire, reaching statistical significance in the women pre-cART and men on cART groups (Table 4). The proportion of women and men reporting genital symptoms in the last 6 months significantly declined in all three groups (women pre-cART 43% to 28%, *p* = 0.03; women cART 51% to 28%, *p* < 0.01; and men cART 34% to 13%, *p* < 0.01). Finally, the proportions of women seeking STI treatment in the last 6 months (both groups), with an abnormal pelvic exam finding (both groups), with an abnormal bimanual exam (women on cART only), and with a clinical diagnosis made after examination (women pre-cART only) declined.

## 4. Discussion

In the SEARCH study, drastic HIV plasma viral load reductions were achieved rapidly, accompanied by slow increases in median CD4 counts over time, similar to what has been found in many other studies in sub-Saharan Africa [6–8]. Reassuringly, the reporting of most cART side effects peaked to at most 20% of patients in the first month of cART, and declined thereafter without intervention. Most patients reported improved appetite, weight gain, and reduced feelings of depression after cART initiation. The proportion of patients on cART with clinically assessed sensory neuropathy increased from 18% at baseline to 27% at month 12, but declined thereafter to background levels. Comprehensive data on systematically assessed side effects in sub-Saharan Africa are scarce, but the published data are in agreement with our data [25–27].

While most of the SEARCH findings are encouraging, some are worrisome, especially in light of limited availability of second and third line antiretroviral drugs: virological failure after 12 months of cART was 11%, 21% of the patients required a drug change due to side effects during follow-up, and about half of the women and 20% of the men continued to feel depressed during follow-up. We reported earlier that cART adherence in the SEARCH cohort was lowest during the first 3 months of cART [18]. While we did not identify any direct statistical associations between side effects, depression, and cART adherence (data not shown), it seems likely that the Rwandan HIV program would benefit from interventions that help HIV patients cope with these factors, especially in the first few months after cART initiation. When patients understand that the majority of side effects are transient and learn how to manage the temporary discomfort (as well as other aspects of introducing cART into their lives), it might be possible to reduce the number of drug switches due to side effects and to improve adherence. The high level of depression in people living with HIV, even when they receive cART, has also been found in other studies [28] and has been recognized by the Rwanda government: the Rwanda National Strategic Plan for HIV (NSP) 2013–2018 calls for improved mental health services for people living with HIV [29].

About one-third of pregnancies in both groups of women before and after HIV diagnosis were unintended, and cART use was not a significant predictor of pregnancy incidence. These findings are in agreement with data from other countries in sub-Saharan Africa [15, 17, 30]. In both groups, condoms were the most frequently mentioned family planning method, as was the case in a recent study in Kenya, Tanzania, and Namibia [31]. The proportion of women using modern contraception other than condoms in the pre-cART group at baseline (50%) was similar to the proportion of currently married Rwandan women using modern contraception other than condoms (42%) in the 2010 Demographic and Health Survey [9]. In contrast, this proportion was only 24% of women initiating cART, perhaps because 49% of them reported not to be currently sexually active. In all groups, contraceptive use declined somewhat in both groups during follow-up despite the fact that the proportions of sexually active women and women trying to get pregnant remained stable in both groups, and the availability of modern contraceptive methods in Kigali increased during the study period [32]. However, these methods were not available in the TRAC-Plus Clinic during the SEARCH study. According to the Rwanda Ministry of Health, only 30% of public clinics countrywide provided integrated cART and family planning services in 2009 compared to 85% in 2011 [33]. Similarly, while only about a third of the female SEARCH participants who carried at least one pregnancy to term since having received their HIV diagnosis reported to have participated in a PMTCT intervention, this proportion would likely be much higher today due to improvements in PMTCT coverage in recent years. The Rwanda Ministry of Health introduced PMTCT option B+ in 2011, and according to them, 86% of public clinics were offering PMTCT option B+ by the end of 2011 [32]. Contraception to prevent unintended pregnancy and PMTCT are both highly effective in preventing HIV infection in neonates. Access to and uptake of family planning and PMTCT services should therefore continue to be closely monitored.

Of most concern are the very high levels of STI coinfections in the SEARCH study. Many patients were coinfected with HSV-2 (79–92%), HR-HPV (38–53%), and bacterial STIs. STI diagnostic testing is not widely available in Rwanda, and many of these infections would likely have remained undiagnosed outside the SEARCH study setting. However, the SEARCH study also showed that STI-related morbidity declined during follow-up, most likely due to access to regular screening and treatment of STIs. In 2011, the Rwandan government launched a nationwide HPV vaccination campaign for girls aged 12–15, which achieved 93% coverage [34]. However, such campaigns do not benefit older women or men, and we therefore recommend regular screening of people living with HIV for cervical and other HPV-related cancers. In addition, we recommend strengthening of STI control in general and continued integration of HIV, STI, and family planning services. We applaud the Rwanda NSP 2013–2018, which states that the Rwanda Ministry of Health aims to increase STI systematic screening from 40% to 75% of people living with HIV, improve treatment of positive cases, and improve reporting of STI indicators [30].

The strengths of the SEARCH study are the comprehensive and systematic assessments of multiple cART clinical and reproductive health outcomes, with up to 24 months of follow-up, and the ability to compare women and men on cART as well as women not yet eligible for cART with women on cART. The main limitation is the fact that follow-up had to be terminated in August 2010 due to cessation of funding, at which time we had not yet achieved 24 months of follow-up for the entire cohort. However, we have no reason to believe that patients who did not complete 24 months of follow-up were systematically different from patients who did.

In conclusion, the SEARCH study showed that the cART program in Rwanda is largely successful, but that cART adherence and clinical outcomes should continue to be monitored and improved. We applaud the significant increase in access to modern methods of contraception, PMTCT services, and HPV vaccination in recent years and recommend that access to, and uptake of, those services continue to be closely monitored as well. Significant work remains to be done to strengthen STI control in Rwanda in general, and in people living with HIV in particular, and we recommend that this work is initiated with urgency.

## Figures and Tables

**Table 1:** Baseline sociodemographic, sexual, and reproductive characteristics.

Characteristic	Women pre-cART *N* = 199^1^	Women on cART *N* = 113^1^	Men on cART *N* = 100^1^
Age in years (median, IQR)^2^	**30 (26–38)**	**37 (31–43)**	37 (33–44)
Highest level of education in years (%)			
No schooling	11.7	8.1	8.3
Some primary/secondary	80.2	73.9	72.9
Secondary completed/postsecondary	8.1	18.0	18.8
Marital status (%)			
Married	58.1	46.9	68.5
Divorced	16.7	13.3	10.9
Widowed	19.2	27.4	7.6
Never married	6.0	12.4	13.0
Number of years when HIV-positive (median, IQR)	2 (0–5)	2 (0–5)	1 (0–3)
Spouse HIV-positive (among married, %)	77.2	77.1	77.2
How do you think you got HIV-infected? (%)^3^			
Sexual contact with spouse	64.1	63.7	**21.2**
Sexual contact with other steady partners	7.1	8.8	**32.3**
Sexual contact with casual/paying partner	17.7	17.7	**42.4**
Other^6^	3.5	10.6	**9.1**
Unknown	7.6	5.3	**1.0**
Number of people to whom the participant disclosed his/her HIV status	2.5 (1–4)	3 (2–5)	2 (1–5)
Age at first sex (median, IQR)	18 (17–20)	19 (17–22)	20 (18–21)
Currently sexually active (%)^2^	**64.0**	**50.9**	66.7
Lifetime number of sex partners (median, IQR)^3^	2 (1–3)	2 (1–3)	**6 (4–11)**
Ever had sex against your will (%)	47.4	53.2	Not asked
Self-identifies as sex worker (%)	3.0	0	NA
Regular alcohol use (%)^3,5^	29.3	25.2	**71.1**
Husband circumcised (%)	34.2	34.6	25.7
Lifetime number of pregnancies (median, IQR)	3 (2–4)	3 (2–4)	NA
Any pregnancy unintended (% of women)	37.8	46.1	NA
Any births ending in abortion, miscarriage, or stillbirth (% of women)	9.4	9.2	NA
Currently using family planning (%)^2^	**65.5**	**48.6**	NA
Condoms^4^	48.8	70.6	
Injectables	27.6	7.8	
Combined oral contraceptive pills	8.7	2.0	
Hormonal implants	8.7	5.9	
Copper IUD	0.8	3.9	
Female sterilization	3.9	3.9	
Periodic abstinence/coitus interruptus/other	8.0	13.7	
Currently breastfeeding (%)	9.3	0	NA
At least one pregnancy since HIV diagnosis (%)	27.6	16.7	NA
Any of these pregnancies unintended (women reporting a pregnancy since HIV diagnosis, %)	36.0	21.6	NA
Ever used PMTCT (women reporting a pregnancy since HIV diagnosis, %)^2^	**42.9**	**20.6**	NA
At least one HIV-positive child (among those reporting children, %)	14.9	10.8	Not asked

^1^At most 5, 4, and 8 missing values per group, except for the number of pregnancies since HIV diagnosis (*N* = 163 and 90, resp.).

^2^Difference between women pre-cART and women on cART is statistically significant at *p* < 0.05 (also shown in bold). At the time of the study, PMTCT option A was being implemented in Rwanda.

^3^Difference between women and men is statistically significant at *p* < 0.05 (also shown in bold).

^4^Among women who reported to use family planning. Women could report more than one method (e.g., hormonal contraception and condoms). No one reported use of diaphragms, male sterilization, or traditional methods.

^5^Regular alcohol use is defined as at least 3 days every week in the last 6 months.

^6^“Other” includes from mother, rape, and other (unspecified). A total of 10 women reported rape during the 1994 genocide.

**Table 2:** Clinical and laboratory characteristics at baseline and during follow-up.

Characteristics	Women pre-cART *N* = 199^1^	Women on cART *N* = 113^1^	Men on cART *N* = 100^1^
Body mass index (median, IQR)—baseline^2^	23.6 (21.3–26.5)	22.8 (20.4–27.3)	21.3 (19.2–22.8)
WHO HIV clinical stage—baseline^2^			
I	84.7	57.4	63.3
II	11.2	26.9	23.5
III	4.1	13.9	11.2
IV	0	1.8	2.0
CD4 T-cell count in cells/*μ*L (median, IQR)—baseline^2^	511 (414–643)	230 (162–316)	220 (137–279)
CD4 T-cell count in cells/*μ*L (median, IQR)—month 12^2^	523 (407–713)	338 (241–439)	329 (216–435)
CD4 T-cell count in cells/*μ*L (median, IQR)—month 24	341 (292–522)	332 (251–474)	364 (233–436)
Plasma HIV RNA concentration in log_10_ copies/mL (median, IQR)—baseline^2^	3.7 (2.9–4.4)	4.7 (4.0–5.2)	4.9 (4.4–5.4)
Plasma HIV RNA concentration in log_10_ copies/mL (median, IQR)—month 12^2^	3.7 (2.8–4.3)	Undetectable	Undetectable
Plasma HIV RNA concentration in log_10_ copies/mL (median, IQR)—month 24^2^	3.9 (3.1–4.7)	Undetectable	Undetectable
Positive urine pregnancy test (%)—baseline^3^	0.5	5.4	NA
Positive HSV-2 serology (%)—baseline	85.9	91.7	79.0
Positive *Neisseria gonorrhoeae* PCR (%)—baseline	14.0	7.3	Not done
Positive *Chlamydia trachomatis* PCR (%)—baseline	4.5	4.9	Not done
Positive syphilis serology (%)—baseline	2.5	3.6	2.1
Any high-risk or low-risk HPV (%)—baseline	48.8	61.2	67.9
Any high-risk HPV (%)—baseline	38.4	53.1	43.2
Incidence of pregnancy (per 100 PY, 95% CI)	11.3 (6.8–18.7)	8.0 (4.3–14.9)	NA
Incidence of *Neisseria gonorrhoeae* (per 100 PY, 95% CI)	12.5 (4.7–33.0)	6.7 (2.5–17.9)	Not done
Incidence of *Chlamydia trachomatis* (per 100 PY, 95% CI)	4.8 (1.2–19.0)	6.6 (2.5–17.5)	Not done
Incidence of syphilis (per 100 PY, 95% CI)	6.0 (3.0–12.0)	6.5 (3.1–13.6)	Not done

^1^Actual sample sizes per variable at baseline ranged from 187 to 199, 102 to 110, and 91 to 100, except for *Neisseria gonorrhoeae* and *Chlamydia trachomatis* PCR (*N* = 157 in women pre-cART; *N* = 113 in women on cART, and this test was not done in men). Actual sample sizes at month 12 ranged from 80 to 85, 85 to 86, and 71 to 74. Actual sample sizes at month 24 ranged from 9 to 12, 42 to 44, and 27 to 29.

^2^The difference between women pre-cART and women and men on cART combined is statistically significant at *p* < 0.05.

^3^The difference between women pre-cART and women on cART is statistically significant at *p* < 0.05.

**Table 3:** Proportion of women and men on cART with sensory neuropathy or perceived fat redistribution during follow-up.

Assessment	Baseline	M6	M12	M18	M24
Clinical diagnosis of sensory neuropathy (%)^1,2^					
Women pre-cART	9.1	4.9	7.7	0	8.3
Women on cART	20.0	20.0	30.1	14.9	4.8
Men on cART	15.5	20.7	22.7	12.7	2.9
Overall perceived weight (% reporting weight gain/no change/loss)^3,4^					
Women on cART	10/51/38	29/49/22	36/43/21	39/36/25	26/49/26
Men on cART	5/46/49	40/42/18	34/52/13	30/44/26	26/51/23
Perceived fat redistribution by body site (% reporting fat gain/no change/loss)^4,5^—women on cART					
Face	5/61/34	66/14/20	77/7/16	65/7/28	79/9/12
Back of neck	67/30/3	76/11/13	85/5/10	76/6/18	81/12/7
Front of neck	64/32/4	73/13/15	86/6/8	74/9/16	81/12/7
Buttocks	59/37/4	73/13/14	79/9/12	65/10/25	79/12/9
Waist/belly	63/29/7	63/12/25	68/5/27	64/11/26	70/12/19
Breasts	70/25/5	76/8/17	79/4/17	82/8/11	81/9/9
Perceived fat redistribution by body site (% reporting fat gain/no change/loss)^4,5^—men on cART					
Face	6/51/43	70/10/21	80/4/16	73/16/11	77/11/11
Back of neck	64/31/5	78/5/16	91/3/6	80/15/5	89/3/9
Front of neck	65/31/4	79/6/16	91/1/7	76/16/7	89/3/9
Buttocks	65/31/4	79/5/15	87/4/9	78/13/9	91/3/6
Waist/belly	51/45/4	66/8/26	77/7/16	65/20/15	83/9/9
Breasts	70/25/6	80/4/15	91/3/6	80/10/10	89/9/3

^1^ *N* = 186, 162, 65, 23, and 12 for women pre-cART and 202, 182, 149, 122, and 77 for women and men on cART. Final diagnosis is based on completing a standardized sensory neuropathy assessment form. Symptoms were current symptoms at baseline and symptoms since the last study visit during follow-up. Vibration sensation was assessed with a 128 Hz tuning fork placed on wrists and interphalangeal joint of great toes. Achilles tendon reflexes were assessed using a reflex hammer. A clinical diagnosis of neuropathy was made if the patient reported one or more symptoms and had uni- or bilateral reduced ankle reflexes or had uni- or bilateral reduced vibration sensation.

^2^In binomial generalized linear models, a neuropathy diagnosis was statistically significantly more likely (at the *p* < 0.05 level) in women and men on cART than women pre-cART, but there were no differences between women and men on cART. The reduction over time in the cART groups was also significant at the *p* < 0.05 level.

^3^ *N* = 107, 97, 80, 67, and 43 for women on cART and 94, 89, 67, 54 and 35 for men on cART. These assessments were not done in women pre-cART.

^4^Based on self-reported weight or fat gain/no change/loss using a standardized questionnaire. Weight and fat gain/no change/loss was reported over the last 3 months at baseline and during follow-up since the last study visit (typically also 3 months).

^5^ *N* = 108, 101, 82, 67, and 43 for women on cART and 95, 91, 70, 55, and 35 for men on cART.

**Table 4:** Behavioral and clinical changes over time by study group.

	Women pre-cART		Women cART		Men cART	
	Baseline	M12	Baseline	M12	Baseline	M12
Living with spouse (%)	58.0	55.0	46.9	39.8	62.6	70.0
Number of sex partners at the last 6 months (median, range)	1 (0–9)	1 (0–2)	1 (0-1)	0 (0-1)	1 (0–20)	1 (0–3)
Currently sexually active (%)	64.0	56.7	50.9	43.6	66.7	71.0
Desire to get pregnant (%)	11.1	11.3	13.2	8.0	NA	NA
Currently trying to get pregnant (%)	9.5	9.7	10.8	5.0	NA	NA
Currently using family planning (%)^1,2^	**65.5**	**49.3**	48.6	36.8	NA	NA
Frequent vaginal sex in last month (median, range)^1,2^	2 (0–7)	2 (0–4)	1 (0–5)	1 (0–6)	**2 (0–12)**	**3 (0–18)**
Used a condom during last sex act (%)	40.6	44.4	43.1	41.8	61.2	76.5
Frequent washing in vagina last week (median, range)^1^	**7 (0–28)**	**7 (0–30)**	7 (0–35)	7 (0–21)	NA	NA
Feeling depressed in the last 4 weeks (%)^1^						
Never/almost never	46.5	55.1	**34.8**	**53.7**	**56.6**	**80.0**
Sometimes	28.3	30.4	**29.5**	**26.8**	**31.3**	**11.4**
Often	25.2	14.5	**35.7**	**19.5**	**12.1**	**8.6**
Sexual desire in the last 4 weeks (%)^1^						
A lot less desire	41.1	37.7	41.1	41.5	**24.2**	**11.4**
A little less desire	24.4	13.0	19.6	9.8	**27.3**	**21.4**
The same or increased desire	34.5	49.3	39.3	48.7	**48.5**	**67.2**
Any genital symptoms in the last 6 months (%)^1,3,4^	**43.2**	**27.5**	**51.3**	**28.4**	**34.0**	**12.9**
Sought STI treatment in the last 6 months (%)^1^	**25.4**	**10.3**	**29.8**	**15.2**	13.1	8.6
Any abnormal pelvic exam finding (%)^1,5^	**53.6**	**28.3**	**60.9**	**39.7**	NA	NA
Abnormal bimanual exam (%)	7.4	13.3	7.9	19.4	NA	NA
Clinical diagnosis made after pelvic/bimanual exam (%)^6^	28.6	18.6	27.3	29.2	NA	NA

^1^Difference between baseline and month 12 shown in bold is statistically significant at *p* < 0.05.

^2^Including the family planning methods listed in Table 1. The proportion of women using specific methods over time did not change, but women pre-cART were statistically significantly more likely to use hormonal methods of contraception than women on cART, whereas women on cART were statistically significantly more likely to use condoms.

^3^For women, these included (all participant-reported, from most frequently reported to less frequently reported) vaginal itching^*∗*^, vaginal burning^*∗∗*^, unusual vaginal discharge^*∗∗∗*^, abdominal pain, discomfort when urinating^*∗∗∗*^, rash external genitalia^*∗∗∗*^, unusual vaginal odor^*∗∗∗*^, vaginal pain^*∗∗∗*^, pain during sex, ulcers, abnormal menstrual bleeding^*∗∗∗*^, and other. The symptoms indicated with *∗* were statistically significantly less prevalent at M12 than baseline in both study groups, *∗∗* in the pre-cART group only, and *∗∗∗* in the cART group only.

^4^For men, these included (all participant-reported, from most frequently reported to less frequently reported): genital itching^*∗*^, genital rash, penile pain^*∗*^, genital ulcers, discomfort when urinating, genital burning, pain during sex, and other. The symptoms indicated with *∗* were statistically significantly less prevalent at M12 than baseline.

^5^These included (all clinician-observed) palpable inguinal lymph nodes, lesions on the external, vaginal or cervical epithelia, unusual vaginal discharge, or abundant cervical mucus.

^6^Including (in decreasing frequency) vaginal discharge syndrome, cervicitis, pelvic inflammatory disease, genital warts, genital herpes, and other.
